# Women’s sexual scripting in the context of universal access to antiretroviral treatment—findings from the HPTN 071 (PopART) trial in South Africa

**DOI:** 10.1186/s12905-021-01513-z

**Published:** 2021-10-24

**Authors:** Lario Viljoen, Graeme Hoddinott, Samantha Malunga, Nosivuyile Vanqa, Tembeka Mhlakwaphalwa, Arlene Marthinus, Khanyisa Mcimeli, Virginia Bond, Janet Seeley, Peter Bock, Richard Hayes, Lindsey Reynolds, HPTN 071 (PopART) study team

**Affiliations:** 1 Desmond Tutu TB Centre, Department of Paediatrics and Child Health, Faculty of Medicine and Health Sciences, Stellenbosch University, Cape Town, South Africa; 2 Department of Sociology and Social Anthropology, Stellenbosch University, Stellenbosch, South Africa; 3 AIDS and Society Research Unit, Centre for Social Science Research, University of Cape Town, Cape Town, South Africa; 4 Zambart, School of Public Health, Ridgeway Campus, University of Zambia, Lusaka, Zambia; 5 Department of Global Health and Development, Faculty of Public Health and Policy, London School of Hygiene and Tropical Medicine, London, UK; 6 MRC Tropical Epidemiology Group, London School of Hygiene and Tropical Medicine, London, UK

**Keywords:** South Africa, Sexual scripting, HIV, Universal test and treat, Treatment as prevention

## Abstract

**Background:**

HIV treatment-based prevention modalities present new opportunities for women to make decisions around sex, intimacy, and prevention. The Universal test and treat (UTT) strategy, where widespread HIV testing is implemented and all people with HIV can access treatment, has the potential to change how sex is understood and HIV prevention incorporated into sexual relationships. We use the frame of sexual scripting to explore how women attribute meaning to sex relative to UTT in an HIV prevention trial setting. Exploring women’s sexual narratives, we explored how HIV prevention feature in the sexual scripts for women who had access to UTT in South Africa (prior to treatment guideline changes) and increased HIV prevention messaging, compared to places without widespread access to HIV testing and immediate access to treatment.

**Methods:**

We employed a two-phased thematic analysis to explore longitudinal qualitative data collected from 71 women (18–35 years old) between 2016 and 2018 as part of an HIV prevention trial in the Western Cape Province, South Africa. Of the participants, 58/71 (82%) were from intervention communities while 13/71 (18%) lived in control communities without access to UTT. Twenty participants self-disclosed that they were living with HIV.

**Results:**

We found no narrative differences between women who had access to UTT and those who did not. HIV and HIV prevention, including treatment-based prevention modalities, were largely absent from women’s thinking about sex. In their scripts, women idealised romantic sex, positioned sex as ‘about relationships’, and described risky sex as ‘other’. When women were confronted by HIV risk (for example, when a partner disclosed his HIV-positive status) this created a point of disjuncture between this new perception of risk and their accepted relationship scripts.

**Conclusion:**

These findings suggest that HIV-negative women did not include their partners’ use of antiretroviral therapy in their sexual partnership choices. For these women, the preventive benefits of UTT are experienced passively—through community-wide viral suppression—rather than through their own behaviour change explicitly related to the availability of treatment as prevention. We propose that prevention-based modalities should be made available and supported and framed as an intervention to promote relationship well-being.

## Background

More than 20% of the 37 million people living with HIV (PLHIV) worldwide reside in South Africa, where there are reportedly nearly 8 million cases [1, 2]. In this sub-Saharan African country, HIV transmission occurs mainly through heterosexual sex and women are disproportionately affected—prevalence among people aged 15–49 years is estimated at 26% for women compared to 15% for men [1, 3].

Globally, HIV prevention strategies have predominantly been targeted at disrupting transmission through behavioural interventions, including abstinence and condom use [4]. The range of prevention modalities has expanded over the past two decades to include a number of biomedical approaches, including HIV ‘treatment as prevention’ (TasP) [5]. This approach is based on evidence from several studies that have shown that PLHIV are unlikely to transmit HIV to their sexual partners if they are virally supressed through effective treatment [6]. TasP has been operationalised on a wider scale through a strategy known as universal test and treat (UTT). UTT entails two components: widespread HIV testing, often at community level, and the offer of immediate access to antiretroviral therapy (ART) for all PLHIV. In addition to offering clinical benefits of ART for PLHIV, the premise is that all people (universally) know their HIV status (testing), and all people with HIV are on ART (treatment). If this could be achieved, then the likelihood of onward transmission would be dramatically reduced, leading to much lower incidence [7, 8].

Since 2015, the World Health Organisation (WHO) changed their recommendations on HIV treatment to encourage countries to adopt the UTT approach [9]. In addition, since 2017, the U = U (undetectable viral count equals untransmittable HIV) movement in Europe and North America [10] has gained traction, through which activists and scientists have raised awareness about the definitive scientific evidence on the preventative properties of ART, focusing on how it enables PLHIV to protect their sexual partners from HIV through treatment [6]. In sub-Saharan Africa, however, studies have shown that TasP messaging has not yet had wide resonance or uptake locally [11, 12].

Despite the scientific consensus on the preventive benefits of HIV treatment, some researchers have posited, however, that the implementation of UTT, and TasP messaging promoting awareness of its benefits, could lead to ‘sexual disinhibition’, whereby people are more likely to take greater risks in their sexual lives [13], although evidence to support this argument is lacking [14]. In this study, we aimed to explore how sexual decision making is shaped by UTT in communities where the approach was implemented.

Decisions around sex are complicated by gender roles, relationship dynamics, and child-bearing intentions [15]. One approach to interpret sexual behaviour that helps us to consider sexual decision-making is sexual scripting. Sexual scripting is described as the accepted sexual norms that are supported, internalised, and endorsed by individuals through the processes of socialisation [16]. In our study, we explored and compared the sexual scripts of young women (aged 18–35) living in communities in the Western Cape province of South Africa where the HPTN 071 (PopART) HIV prevention trial offering UTT was implemented [17].The intervention implemented between 2013 and 2018, included expanded HIV testing and counselling services at household level, and access to treatment for all PLHIV. The trial setting therefore allowed for an early exploration of community responses to HIV treatment as prevention, before this approach was implemented more broadly.

In this study we aimed to understand how TasP is incorporated in women’s sexual behaviour decision-making in a high HIV-burden and generalised epidemic setting. In the analysis for this manuscript, we focussed on intrapsychic scripts—or women’s “personal sexual cultures” [18]—to explore how meaning is attributed to sex relative to UTT. We found that neither TasP nor UTT featured in women’s sexual decision-making processes. Women tended not to focus on HIV prevention in their sexual scripts, but idealised romantic sex, and positioned ‘risky sex’ as belonging to transgressive ‘others’.

### Women, sex, and HIV prevention

To contextualise our findings, we provide an overview of representations of women in HIV research in Africa. As HIV took hold of the continent from the 1990s, an expanding body of work developed with a focus on sex and how it fuelled the epidemic [19]. In the early years, Packard and Epstein [20] documented important similarities between research on HIV, tuberculosis, and syphilis, showing how the ‘unhealthy behaviours’ of people living in Africa were often deployed as a central explanatory framework, while environmental, social, and structural factors were overlooked. Thirty years later, the framing of Africa as a ‘problem area’ and those living in Africa as hyper-sexual continues to shape how HIV is researched [21, 22]. Thomas and Cole [23] describe how this extensive focus on HIV transmission in research in/on Africa has reduced understandings of intimacy in Africa to a concern with sex and risk, while emotional connections have been sidelined.

Young women are often included as priority groups in HIV research because they are described as particularly vulnerable to HIV due to both biological and social determinants [24–26]. In the public health discourse, sexual behaviour of women in Africa continues to be framed around one-dimensional concerns such as violence and abuse [27], transactional sex [28], and reproductive issues [29], with limited focus on intimacy, interpersonal connections, pleasure, or the internalised understandings of sex. However, as Ruark [30] notes, in southern Africa, these issues often coexist with, rather than oppose, intimacy and love in relationships. Disparate patriarchal power dynamics, gender inequality, violence, male dominance, and sexual coercion are often part of complex understandings of intimacy and sex [30, 31].

With the roll-out of extended testing initiatives and treatment-based prevention modalities on a wider scale through the strategy of UTT, there has been speculation about the potential effects of these prevention technologies on women’s understandings of (safe) sex and their sexual behaviours. In a separate analysis from research in the HPTN 071 (PopART) communities prior to the implementation of the intervention, community members reported generally favourable experiences and expectations of ART for treatment purposes, but expressed hesitancy or uncertainty in relation to the preventative capabilities of ART [11]. Nonetheless, fears of sexual disinhibition have resurfaced [13], while others have posited that UTT might lead to the normalisation of HIV where ART is incorporated into routine dialogues on sex—in communities and between intimate partners [32]. Through sexual scripting, we explored women’s narratives about sex and investigated how prevention modalities featured in these scripts.

### Sexual scripting and HIV

Sexual scripting is a framework where the metaphor of ‘scripts’ is used to understand the mutually shared conventions that guide individuals in their engagements with sexual interactions [33, 34]. Individuals are responsive to each other’s cues in these exchanges, and react according to broader accepted social or cultural norms and expectations [35].

According to Simon and Gagnon [16], scripts develop through the interactions between three distinct spheres of human experience: cultural scenarios, interpersonal scripts, and intrapsychic scripts. Cultural scenarios are socially determined normative expectations and provide the guidelines by which sexual interactions are expected to take place. These scenarios determine, for instance, the appropriate time, place and form of sexual expression [36]. Individuals employ sexual scripts to align their behaviours with cultural scenarios. Interpersonal scripts evolve when pairs or groups adapt generic normative scripts from ongoing experiences and interactions with each other.

At the most intimate level, the intrapsychic script is the mechanism whereby individuals attach and internalise ‘meaning’ to sexual behaviour as they interact with the social world over their life course [18]. It is at this level where motivational elements are located that allow individuals to commit to a particular sequence of events [33]. The distinction between levels of scripts is conceptually useful to explore multiple personal, interpersonal and social influences on people’s thinking about sex. However, ‘levels’ to the scripts should not be reified as all are experienced simultaneously and indivisibly.

Wiederman [37] suggests that scripts are used to interpret “what is considered normative within a culture … providing directions for how to feel, think, and behave in particular situations”. Sexual behaviour is a process that entails the acceptance of a socially defined performance and the development of personal meaning attached to this performance.

Researchers have employed scripting to interpret sexual behaviour in relation to HIV in Africa, in which include studies on concurrent sexual relationships [38], with young people [39, 40], and with heterosexual couples in trial settings [41]. Scripting has also been used to understand sexual behaviour amongst vulnerable women [33, 35, 42, 43]. With the evolving HIV prevention landscape, scripting can be employed as a means to understand if and how prevention modalities are incorporated into sexual scripts. Through intrapsychic scripts specifically, we can explore how internalised interpretations of sex can shape (and are shaped by) HIV prevention methods.

Researchers have acknowledged the role of gendered scripts in determining sexual behaviour and access to HIV prevention modalities [33]. For instance, in their study amongst African-American women in Washington DC, Bowleg and colleagues [42] found that, as part of accepted sexual scripts, men generally had more control than women in relationships—both in terms of sexual activity and condom use. In these accepted scripts, where women lack control, women’s risk of HIV may be indirectly or directly increased. Similarly, in their study on urban women in the USA, Ortiz-Torres and colleagues [35] concluded that most women's narratives continued to endorse the traditional gender script of men as initiators of sexual encounters and women as submissive partners, while in South Africa, Duby and colleagues [41] described how the interpersonal scripts of women tended to overemphasise the satisfaction of male partners.

While some studies focused on HIV prevention in sexual scripts [33, 44], less attention has been paid to how women’s conceptualisations of HIV risk shape their sexual scripts [45]. In our analysis below, we explore young women’s sexual narratives to understand how HIV prevention feature in intrapsychic sexual scripts in the context of UTT, when compared to places without widespread access to HIV testing and immediate access to treatment.

## Methods

We analysed data collected as part of the HPTN 071 (PopART) trial conducted in 9 peri-urban communities in the Western Cape Province of South Africa and 12 communities across Zambia. This three-armed community randomised trial was aimed at assessing if the implementation of a community-based HIV prevention intervention would reduce HIV incidence at a population level [17]. In the intervention arms (Arms A and B), communities received door-to-door HIV testing, referrals for medical male circumcision, condom distribution, TB and STI screening, and referrals to local health facilities for treatment for PLHIV from community based health workers [46]. As part of the intervention, people living in Arm A communities received UTT (and access to TasP) from the start of the trial. In addition to providing health services, referrals and counselling, the community health workers were tasked with explaining the intervention and the overall aim of the trial to community members, including the preventative ability of HIV treatment and the benefits of UTT. Control communities (Arm C) received health services according to standard of care. Changes in national treatment guidelines in South Africa allowing all PLHIV to access HIV treatment effectively meant people living in Arm B communities also received UTT from 2016.

Approximately 2000 individuals per community were randomly selected for cohort follow up to measure the primary trial outcome. The trial results showed an overall reduction of ~ 20% in HIV incidence in intervention arms (Arms A and B) compared to control communities [7].

As part of the trial, we conducted longitudinal research with a cohort of 89 households (⁓ 290 individuals) in the South African sites over 18 months between 2016 and 2018. We conducted a series of interactive and ethnographically informed interviews structured around ‘domains of life’, focussed on: household composition; place, space and movement; how people ‘get by’; love, sex, and romance; healthcare behaviour; and hopes, fears and ambitions [47].

Discussions were held with either groups or individual household members, depending on the topic and participant availability. Participants were recruited during community observations, recruitment drives, and through referrals from health workers. Households were recruited through targeted recruitment drives and through snowball sampling for at risk populations (e.g. sex workers, men who have sex with men). We purposively sampled households across trial arms to ensure diversity in terms of age, gender, household structure and location within the community [48]. We recruited households from all 9 South African study communities and ensured that at least half of all households had one or more member who self-reported living with HIV. To document intervention implementation, we overrecruited households from intervention communities, ~ 4:1 intervention:control. We worked in teams and were paired to ensure a good ‘fit’ (language, age or gender compatibility) with participants [47].

Discussions were designed to be more than one-off conversations. We embraced the principle of ‘deep hanging out’ where researchers use participatory observations to immerse themselves in the social experience of participants at an informal level over extended time periods [49]. We had multiple (7–12) interactions with households, spending between 1 and 3 hours per discussion with participants.

Discussions on sex were structured along three parts: an open conversation about community beliefs around sex; a timeline and details of previous relationships; and the intimate and detailed ‘story’ of participants’ most recent sexual encounters. We probed participants’ experiences related to HIV (prevention and disclosure) only after the open-ended discussion. To mitigate social desirability during fieldwork, these conversations took place after more than 6 months of regular rapport-building. To make it easier to describe intimate acts, participants were given the option to speak directly to the audio recorder while the researcher stepped away, although very few participants (< 10) opted to do so.

Reliable, accurate and consistent data on sexual behaviour is notoriously difficult to collect due to the social desirability and the (still) taboo nature of the topic [50]. To ensure that researchers were comfortable facilitating open and inclusive discussions on the topic, we conducted workshops and practiced several iterations of the open-ended discussion guides. Researchers were trained on appropriate and ethical responses when encountering reports of violence, abuse, drug use, and unsafe sex in discordant relationships in the field. After discussions, researchers were instructed to provide accurate health information and, where needed, to refer participants to appropriate support structures (like social services).

Interviews were conducted in English or another local language (Afrikaans, Xhosa). All discussions were recorded, transcribed verbatim, translated to English and anonymised, mostly by the researchers conducting the interviews. Transcriptions included detailed notes, including on, for instance, tone and hesitations, and translations included notes on meaning of specific translated terms, including metaphors and imagery used.

Pseudonyms are used throughout for reporting. The first and several of the supporting authors contributed to data collection.

For this analysis, we included data from all women aged 18–35 years-old (given younger women’s higher HIV risk) who participated in individual discussions for the research module on love, sex, and romance, and who self-reported that they had sex in the past 12 months. We excluded women who self-disclosed that they were engaging in sex work at the time of the interview and women who are healthworkers, as these groups had different experiences of sexual relationships or health knowledge that deserve separate analyses. From the 89 housholds, the sample included 71 women (with 20 self-disclosed PLHIV), including 58 in intervention communities (Arm A, n = 49; Arm B, n = 9) and 13 in control communities (Arm C).

We employed a two-phased thematic approach for the analysis. In phase one, co-authors (LV, SM, TM, KM, AM, NV) identified all extracts with key topic areas, which include: participant definitions of ‘sex acts’; detailed sex act descriptions; HIV prevention strategies; contraceptives; HIV risk perceptions; and relationship dynamics. In phase two, we conducted a thematic comparative analysis, as described by Braun and Clark [51] to group and compare the sexual scripts of women in intervention communities to women in control communities. Our analysis focused in particular on women’s intrapsychic sexual scripts.

The HPTN 071 (PopART) trial received ethical clearance from the London School of Hygiene and Tropical Medicine and the Stellenbosch University research ethics committee (N12/09/056; N12/11/074). All participants signed written informed consent prior to participation and ongoing consent was confirmed during follow-up discussions.

## Results

In this analysis of the narratives of the 71 women participating in discussions, we found no noticeable differences in the sexual scripts of women by trial arm. For most women (including both those living with HIV and not), HIV remained largely unacknowledged in their described sexual experiences. When asked about ‘prevention’, women referred almost exclusively to contraceptive methods and researchers had to probe to elicit responses specifically related to HIV, with HIV prevention descriptions limited to condom use. When the team asked about biomedical prevention modalities, including TasP, no participants reported hearing of these options before. All participants in intervention communities had received the door-to-door testing services but did not relate this to TasP. Rather, the intervention was perceived as an effort to expand access to HIV testing. In addition, generally people believed in the therapeutic benefits of ART, and, even after being informed of TasP, somewhat hesitant about but its prevention benefits.

More broadly, we identified three dominant framings of women’s intrapsychic sexual scripts: (1) the idealisation of romantic sex; (2) the conceptualisation of sex as being about relationships; and (3) the positioning of risky sex as ‘other.’ HIV and ‘prevention’ were framed as challenges to normative sexual scripts. Finally, we demonstrate specific points of disjuncture in women’s intrapsychic scripts, where women had to manage the imposition of HIV, including risk perceptions, in the ‘meanings’ they attached to sex.

### Sexual scripts and the silence around HIV

We found that spontaneous references to HIV and risk were mostly absent in the narratives of women, especially for women who were not living with HIV. Women’s descriptions of their sexual interactions with their intimate partners seldom included HIV prevention methods. When probed on ‘protection’ during sex, women readily referred to contraceptive methods, including the Depo-Provera injection, contraceptive implants, or occasionally the “pulling out” method, rather than HIV prevention. Pregnancy prevention was framed as women’s responsibility with few men reportedly engaging with their partners on birth control.

Nita (20, Arm B), had been diagnosed with HIV a few months prior to our discussion. She had a casual relationship with her partner, whose HIV status was unknown to us. Nita told us that she ‘sometimes’ used protection. When we asked why, she explained:

**Table:** 

Nita	It is when I think that my contraceptive [injection] is finished [needs to be replaced]
Researcher	Then what does [your partner] say when you are taking [condoms] out?
Nita	I give it to him and he doesn’t say anything, I tell him that I don’t have contraceptives, and I don’t want a baby

While Nita did occasionally incorporate condoms as part of her sexual interaction, it was not for the purpose of HIV prevention for her partner, but rather for pregnancy prevention, as gendered cultural norms often dictate women do. When probed about HIV prevention specifically, descriptions of biomedical modalities were wholly absent in the women taking part in this study’s narratives. None of the participants mentioned treatment-based options as HIV prevention options. After we described the potential of ART as a means to viral load supression and to prevent onwards HIV transmission, none of the women indicated that they had heard of this message. This despite the participants living in communities where HIV testing and referral for ART (and TasP) had been offered door-to-door and annually for each of the preceding 2–3 years and government HIV treatment guidelines changed to allow all PLHIV treatment since 2016. When we described the concept of TasP to Zinhle (19, HIV negative, Arm B), she had several questions on viral suppression. She told us she had never used condoms with her boyfriend of the past 4 years, Robert, and expanded:

My boyfriend is positive, but we tested, three times, I’m negative … We went to the clinic last month … because I saw that he was sick. I told him, ‘Baby, you are taking treatment and you are hiding [things] from me’. He told me, ‘Baby I’m [HIV] positive’. ‘Okay fine’, I said to him, ‘let’s go to the clinic’ … I was negative, he was positive.

Zinhle explained that she anticipated that she would receive a positive HIV test because she had had sex with Robert who was positive—she assumed transmission was automatic and immediate. Her confusion about her repeated HIV negative test results illustrated that, while the couple used no other form of prevention, they were unaware of the potential of HIV treatment as an effective prevention strategy. Although Zinhle was concerned with Robert’s physical health, she did not frame this concern as connected to their sexual script.

Chantelle (31, Arm A), who was living with HIV, was also in a sero-discordant relationship and had disclosed her status to her long-term friend and then partner, Kyle. When asked explicitly about HIV prevention, she reported that she always insisted on using condoms, but that they had recently started discussing having children together. Although Chantelle had heard that it was possible to become pregnant without transmitting HIV to her partner, she was unsure of what they should do to make this happen:

He asked me if maybe, [we could] have a baby. I said that when the time comes the two of us need to go to the clinic … He also wants to know how [it works]. I don’t know either … We have to go to the clinic and find out about condoms and so on. I don’t know, because when a child gets made, then the condom has to be gone! I can’t give him an answer.

Most women reported occasional condom use during sex, while few told our research team that they insisted on consistent condom use with intimate partners. A few women also presented HIV testing, including proxy testing (where a sexual partner’s HIV test result is adopted or assumed as one’s own without confirmation through testing oneself) as a way to mitigate HIV risk without taking other steps to counter potential HIV transmission, which might have jeopardised the continuation of the relationship. Regular testing was seen as a protective measure, one that many women welcomed. Landi (33, Arm A) who was in a longterm relationship with her partner, Timo, declared that she was HIV negative. When we enquired about Timo’s status, she explained:

He is too, because the two of us, how can I say, if I test myself, obviously the two of us sleep together. If we have sex tonight, he climaxes, the two of us both [climax]. I will get that ailment [HIV] from him and he will get the ailment from me … He doesn’t test himself, but I do it. If I go again to get myself tested and they say I have the illness [HIV], obviously who does it come from?

Landi’s relationship with Timo also illustrates the gender-related nature of care and the responsibility of managing health for people in intimate relationships. Landi, as with many other women, takes on the role of the partner who receives the HIV test and confirms the joint status of the couple.

Rosalie (33, HIV negative, Arm A) told us that she tested with her partner every month. When asked why they tested so often, she explained, “because, around here, a person doesn’t know … We share toilets [communal ablutions]. Things like infections and stuff can come”. She also described that she was not worried about her partner “messing around”, because “he made a promise to me and I need to trust him”. For Rosalie, HIV risk is not located in the domain of her intimate relationship. As part of her intrapsychic script, or the ‘motivational elements’ [33] attached to sex, she, like many other women, emphasised the need for ‘trust’ and an unfaithful partner associated with HIV risk was not considered as an option. Rosalie rather deems her relationship as a safe and appropriate space for sex and blames, perhaps as a deflection, communal ablutions as the potential source of HIV. Interestingly, we found that the same participants (like Rosalie) would, in other conversations, readily describe accurate HIV prevention knowledge, but not in their narratives about sex.

### Women’s intrapsychic scripts

While HIV did not feature in the way that most participants constructed their intrapsychic scripts, internalised framings around sex were constructed along the following: (1) the idealisation of romantic sex; (2) sex as being about relationships; (3) and risky sex as ‘other’.

1. The idealisation of romantic sex

Many women defined the act of sex as synonymous with love, trust, respect, and care. This is consistent with romanticised normative scripts described by researchers in other contexts [52, 53]. However, for most of our participants, as with many women in Southern Africa, experiences with intimate partners are often removed from these idealised scenarios, as violence, betrayal and distrust were frequently experienced. Despite these tensions, idealised concepts of sex formed an integral part of the intrapsychic scripts of participants.

Carli (31, HIV negative, Arm C) had been in a relationship with her partner, Ronaldo, for approximately 6 years. In her descriptions of how sex during the early stages of their relationship, Carli employed normative and even stereotypical imagery of romantic sex:

We were very in love. When I got home in the evenings, the roses were scattered on the bed with the red nighty [lingerie] and the chocolates! … And there’s champagne! … [Ronaldo] and I were hungry for each other! They [friends] said … it looks like I can’t wait to get home so I can jump [claps hands for emphasis] on him or he wants to kiss me!

Both Carli and Ronaldo adhered to normative romanticised scripts of sex, and idealised conceptions of sex show how scripts operate across the intrapsychic and cultural spheres. It was also reflected in Carli’s description of the sex act:

[Sex is] not supposed to be with anyone. And the person with who you’re doing it has to respect you. Sex isn’t just for sex. You should know the person and don’t just let him, like “shoot a card” [make sexual advances].

Despite these conceptualisations of sex as respectful and intimate, Carli’s recent experiences were not aligned with these ideals and she acknowledges that there are discrepancies between her internalised expectation and her lived reality. She explained:

Look, I don’t experience it, like I would tell you “it’s fantastic” [claps hands] or “nice” or “I enjoyed it”. To me … it’s a matter of, I have to. It must happen because otherwise I’m going to be bothered for the entire night …You see it’s not the right reasons anymore, to be intimate.

For Carli, sex with Ronaldo was no longer for the ‘proper reason’ of intimacy, but rather a task that needed to be done to avoid being ‘bothered’ by her insistent partner. Carli also described how the relationship became violent: “He hit me a lot, he’s very aggressive, violent, he has probably tried several times to kill me.”

Similarly, Rosalie (33, HIV negative, Arm A) who emphasised the importance of trust in her relationships in the section above, had been with her boyfriend, James (35, HIV negative), for a few weeks when we also met him. They were both regular methamphetamine users. Rosalie would often explain to us that sex and romance was an important part of her life and that she prioritised what she called her weekly ‘mommy’s night’, or date night, where the couple would be intimate. She explained:

If we have sex, I say we make love, or we share each other … Others say ‘fuck’, but that isn’t what you should call it. Because if you really love someone, and you are going to have sex, then you two are sharing each other … You can’t just say you are going to ‘mount’ her; you must say that you are going to make love. [Otherwise] you think nothing of her.

Rosalie describes sex as an act beyond the physical. She emphasised intimacy and suggested that a lack of respectful sex indicates a disregard for your partner. However, while Rosalie prioritised intimacy, her understanding of sex and pleasure was also framed around the needs of her male partner. When asked how important sex is in her life, Rosalie said:

It is very important to satisfy your guy because that is what a man needs. If you can’t satisfy your man, he is going to have a look around to find what he wants. If your guy wants sex, you should never refuse him. You owe him that.

For Rosalie, her intrapsychic sex script was framed around the idealisation of romance, but also the gendered utilitarian function of sex as a means for women to sustain their intimate relationships.

2. Sex as about relationships

We found that sexual narratives were not concerned with the physical health and wellbeing of bodies, but rather about the wellbeing of perceived intimate relationships. Wilma (27, HIV negative, Arm A) explained to us that:

Sex for me, it is part of who I am … because thats how me and my boyfriend now again get to know each other and … I look forward to trying something new [sexually] … [but] God, I am also not in the mood for that anymore ... I just stay so tired .. but sex is part of it [their relationship].

Wilma explained that sex is seen is an essential part of their relationship and maintaining intimacy, regardless of her lack of physical energy.

Even more pointedly, when an HIV diagnosis was revealed in relationship, for many women the primary concern was the interpersonal implications. For instance, Mara (21, LHIV, Arm A) and her boyfriend, Zonke (22), opted to test together for HIV with a team of community health workers at Mara’s house. The couple had been together for a few months and, according to Mara, trusted each other. While Mara received a positive result, her boyfriend tested negative. The unexpected diagnosis meant that the couple had to renegotiate their sexual relationship. Mara explained that she thought she should end the relationship because she feared infecting her partner. Zonke, however, insisted that the relationship should continue. After using condoms once after the positive test result, Zonke told Mara that they should stop using condoms and return to their normal routine of condomless sex.

Since Mara’s diagnosis, she briefly linked to treatment but had since stopped taking ART. When asked about this decision, she replied: “When I’m drinking pills, it’s as if my love would be low on Zonke. Maybe he would be embarrassed”. Mara infers that taking treatment would lower her libido which would challenge her expected role of engaging and enthused sexual partner. This could make Zonke feel less desirable and possibly less masculine, which would lead to embarrassment on his part. The couple briefly considered the implications of Mara’s HIV diagnosis on the future of their relationship and potentially Zonke’s health. However, they soon reverted to established sexual scripts, where condoms did not form part of their sexual interaction. Mara’s concerns about HIV and treatment were not framed around health (her own or Zonke’s), but rather about the implications for her sexual relationship with her partner. Their relationship illustrates the interaction between intrapsychic and interpersonal scripts as the couple confirms their accepted sexual script. Additionally, in this case, both Mara and Zonke dismissed the seriousness of the risk of HIV and preferred to focus on other aspects of the relationship, such as pleasurable sex and intimacy.

3. Locating risk in the ‘other’

When women were asked specifically about HIV, risk was presented as fluid, context-specific, and mostly relevant to ‘others’, often those who were believed to be involved in ‘morally transgressive’ behaviours. ‘Others’ included sex workers and their clients, men who have sex with men, and teenage girls, who were described as easily persuaded to have unprotected sex with older men. While most participants described how they had been sexually active as teenagers and almost half had had children while still in their teens, there was a dissociation between participants’ behaviour and their beliefs about risky ‘others’. Again, intrapsychic scripts and the internalised meaning of sex (appropriate, desired, and acceptable) were in conflict with lived experience.

Cherise (31, HIV negative, Arm A), who had an unplanned pregnancy in her twenties, noted that with “the teenage pregnancies the HIV comes in, because the men make them dumb, they can easily be manipulated.” She added, “every person has his own life and what he does with his life is his business, but if you are begging to get sick, then you should just go to the sex workers.”

Carli (31, HIV negative, Arm C) who, as described in the section above, was in a contentious relationship with Ronaldo, did not consider her relationship ‘risky’. This was despite describing how her partner cheated on her and how she contracted an STI. In her account, she described the other woman as dirty, locating sexual risk as ‘other’:

**Table:** 

Researcher	Have you guys ever used condoms?
Carli	Never during our entire relationship. In the 6 years we have been together we’ve never used condoms
Researcher	Do you think he used condoms with other partners?
Carli	I’ve asked him about that many times. He says yes, but I don’t know. Because the one time when he also had a girl [cheated] and then after a while, he came back. Then we … got together [had sex], then I picked up that thing [STI]. It was almost like the girl was dirty. You see my lower body [vagina] itched very much and burned. I went to get pills, then they [health workers] said the girl was dirty. That’s why she gave him that filthy disease

HIV was placed as belonging outside of accepted intrapsychic scripts, where women understood themselves to engage only in proper and respectable sex [54], and where an association with HIV would cast doubt on the perceptions that they have of themselves [47].

Various participant used observations to reaffirm the position of being ‘safe’ in their relationships. When Viv (28, HIV negative, Arm A) described how she made decisions around sex with her partner, Trevor, she assumed that he was HIV negative because he carried condoms, although she had never used condoms with him. The assumption was that he used condoms with ‘other’ women, and that, because they had a trusting relationship, HIV risk was minimal. She explained: “[We] always had clean [condomless] sex. But I trusted him, and he trusted me. He should know I wouldn’t go to another guy.” In her explanation, Viv does not consider the possibility that Trevor might be unfaithful.

### Points of disjuncture in intrapsychic scripts

In the extracts above, we have shown how HIV prevention, is largely absent in women’s sexual scripts. Most women positioned HIV prevention methods as difficult to incorporate into preferred sexual scripts—much like intimate partner violence or unfaithfulness. When HIV and risk is considered, it was positioned as ‘in tension with’ or as an imposition to accepted intrapsychic scripts.

When prompted to discuss HIV prevention, Rosalie (33, HIV negative, Arm A) had fluid descriptions of her perceived HIV risk. When she was in her teens, Rosalie engaged in sex work, although she rarely spoke about it and did not disclose her past to James. When asked for a timeline of sexual partners, she excluded clients from this period of her life. Her time selling sex was seemingly omitted from her understanding of sex. She also readily stated that she had never used condoms during sex. She considered her stable relationships as safe sexual spaces, and was therefore concerned when her previous partner cheated on her:

**Table:** 

Researcher	Earlier you said that you were worried about HIV. When was this?
Rosalie	It was the time my children’s father was messing around [cheating]
	… I had myself tested and I stayed clean [negative]
Researcher	Have you ever used condoms?
Rosalie	No, never. I am not one for condoms … [reconsiders] I mean, previously, when I was on the street [sex work], then it was important to use them

Rosalie was aware of the protection offered by condom use during transactional sex. However, her intrapsychic understanding of sex with her trusted intimate partner was markedly different from the script related to sex work. Sex with James was, as described above, romantic (‘mommy’s night’, ‘making love’), condomless, and by association, risk free, while sex work was risky and thus required protection. Rosalie positioned her relationships with James as exceptional, allowing (and welcoming) condom-less sex, understanding it to be the “safe” exception.

Others experienced a shift in their perceptions of risk when unexpectedly confronted by a partner living with HIV. Lihle’s (32, HIV negative, Arm A) partner, Nhlobo, had recently disclosed his HIV status to her. The information forced her to reconsider her perceptions of sex, risk, and the relationship:

**Table:** 

Lihle	Last week he told me about his status. I was like, I don’t know if I must continue with that [relationship] or not
Researcher	Have you had sex with him?
Lihle	Not yet. I’m scared. What if we make a mistake, or the condom will break and then I’ll be at higher risk of getting it [HIV]. I’m not sure … because he’s so decent and quiet

Nhlobo’s revelation prompted Lihle to go for an HIV test, which she had not done in more than 2 years:

As soon as I heard his status, I ran [to get] mine … I’m still fine [negative]. But now it seems like I’m taking a risk here with my life. This person is older than me, [he] achieved more things in his life … I’m still confused ... Sometimes I’ll say ‘No, it’s not a problem’. But now it’s practical … I used to tell myself, I’ll [use] condoms. It was a theory [hypothetical] that time, now it’s practical. I have a lot of negative thoughts in my mind … There is risk and then it’s a responsibility. What if we have sex? Then all the time, I must be careful.

Her partner’s revelation meant that Lihle had to reassess her own level of risk, and her potential acceptance of being in a sero-discordant relationship. The act of sex that was safe in one context, was repositioned as risky in another. To manage the disjuncture, Lihle questioned the viability of the sexual relationship as it did not meet her (idealised) expectations. At this moment of rupture, where she is confronted with a reality that challenges her accepted understanding of the meaning of sex meant that she had to rewrite her sexual script—either accepting and including sex with a partner living with HIV as an option, or rejecting a partner based on his HIV positive status and the associated risk.

HIV prevention was positioned outside of intrapsychic scripts about sex, and HIV risk assigned to ‘others’. When women were confronted with the possibility of HIV exposure in their partnerships they managed this—by adjusting their scripts—in three ways: (1) dismissing the seriousness of the risk and focussing on other aspects of the relationship, (2) questioning the viability of the sexual relationship since it did not meet their idealised expectations—although this was limited to their talk about the relationship and we did not find any women to have actioned this talk by ending their relationship; (3) relabelling and making exceptions for some sex acts within relationships.

## Discussion

In our findings, we show that HIV generally does not feature in the sexual scripts of women living in high HIV burden settings in the Western Cape province of South Africa. In their descriptions of sex, most women in our study maintained normative and idealised romantic intrapsychic scripts, and described sex as intimate, respectful, and an act that transcended physical attachment. Women maintained these scripts even when experiencing violence, assault, and unfaithful partners. Continued coherence of a preferred sexual script in an established partnership would be prioritised over physical health, including the prevention of HIV transmission in confirmed sero-discordant relationships. For many women, sex was internalised as a way of sustaining relationships and to ensure that partners were satisfied. Additionally, many of these scripts adhered to gendered norms and expectations, where women anticipated taking on submissive roles, took on the responsibility of pregnancy prevention and care, and prioritised the sexual pleasure of their partners.

We compared the patterns of intrapsychic scripts by arm, but observed no qualitative difference. Further, when explicitly probed on HIV prevention methods, participants only mentioned condoms. Findings from a seperate analysis showed that participants in trial communities had limited awareness of TasP [55]. Similarly, this analysis showed that treatment-based prevention methods and UTT as a strategy to avoid HIV transmission were not acknowledged by any of the women. This is in spite of the active promotion of UTT in communities in the PopART trial and the changes in national HIV treatment guidelines in South Africa in 2016.

Regardless of the dual avenues for women in our study to access and incorporate the message of TasP—either through the door-to-door intervention or through the national HIV services—it was not incorporated into their sexual narratives. Simon and Gagnon noted that for sexual scripts to change, motivation and opportunities are key [16]. These were lacking in the scripts of women in our study. The complexity of internalised sexual meanings, objects, and practices [18] do not currently allow for easy inclusion of prevention as part of the accepted scripts of women in our study context. In other parts of the world, the U = U narrative promoted by activists and endorsed by UNAIDS, has gained traction and has been embraced by PLHIV communities as a liberating and hopeful message that frees intimate relationships from the fear of risk of HIV transmission [6, 10]. From our findings, we show that the message of TasP has not yet filtered through to people in communities most affected by the epidemic. Researchers in other settings have found that the implementation of TasP as an effective HIV prevention method in sero-discordant relationships is dependent on partner dynamics such as status disclosure, safety negotiation, and adherence support, and requires the acceptance of prevention as part of relationship scripts [56]. Our findings suggest that even in the context of UTT, TasP remains dependent on these relationship dynamics. For women in sero-discordant realtionships to capitalise on the benefits of TasP by supporting their partners to be on ART will most likely require specific intervention and support, most likely delivered to couples. Since HIV prevention is difficult for women to internalise as part of sexual scripts, such interventions would need to frame TasP as a means to protect and maintain relationships, rather than as an integral part of the meaning of sex. These efforts would, however, not necessarily benefit women who are in vulnerable relationships. In their study on PrEP and women in South Africa, Hartmann et al. [57] suggested that when PrEP is framed in a positive and empowering way that avoids linking it to relationship risk, it may ultimately encourage greater uptake. Similarly, we propose that prevention modalities, including PrEP and TasP, should be made available and supported, but should be framed as an intervention to promote relationship well-being, not about preventing onward transmission of HIV.

The aggregate reduction in viral burden from UTT is likely to benefit women not living with HIV, but only if men are actively engaged in care and virally suppressed. As it currently stands, men in South Africa are underrepresented in the ‘care cascade’ and are less likely to know their status or be virally suppressed through treatment [1]. Without support structures in place for men, UTT as a truly ‘universal’ mode of HIV prevention that benefits women as well will be difficult to utilise.

One of the main strengths of this analysis is that it is based on a large, diverse sample of more than 70 young women. Further, the longitudinal approach enabled researchers to develop rapport with participants over time which ensured that deeper, detailed discussions on the sensitive topics of sex and HIV could be conducted. The open-ended ethnographic approach allowed for participants to tell their stories without interruption, which allowed for a better understanding of women’s intrapsychic scripts. The diverse team involved in the rigorous analytic process ensured that the data was accurately interpreted. However, a limitation to extrapolation from the findings in the study include that it is a qualitative study with the possibility that it over-represents the experiences of people from key populations, because of the purposive sampling for diversity. The sample included more women from intervention communities receiving the trial intervention of door-to-door testing and access to ART regardless of CD4 count and fewer from the control arm. However, we believe that this imbalance is appropriate given the aim of understanding women’s experiences of sexual scripting relative to the context of treatment as prevention (only available in the intervention communities at the time). More generally, women’s experiences of sex are diverse and shaped powerfully by heterogeneous sociocultural contexts across communities which may differ and transferability of these findings to others in the region are dependent on similarity with these contexts.

In addition, eliciting detailed and honest narratives of sexual behaviour is known to be challenging and social desirability is a concern for many researchers in the field. However, through establishing relationships with participants over a longer time period and building rapport, we were able to counter some of these challenges and to engage in meaningful and open conversations with participants.

## Conclusion

We found that women’s relationships continue to be framed around normative and idealised sexual scripts. Women prioritised the coherence of romanticised sexual script and maintaining established relationships over physical health, including HIV prevention. As UTT is accepted as HIV prevention strategy in South Africa and across the globe, we suggest that further research is needed as to how the expanding set of available modalities are framed when people in relationships make decisions around intimacy, well-being, prevention and health.

## Data Availability

The datasets used and/or analysed during the current study are available from the corresponding author on reasonable request.

## Abbreviations

ART: Antiretroviral therapy
PLHIV: People living with HIV
TasP: Treatment as prevention
UTT: Universal test and treat
U = U: Undetectable viral count equals untransmittable HIV
WHO: World Health Organisation
